# Eye movement responses to health messages on cigarette packages

**DOI:** 10.1186/1471-2458-12-352

**Published:** 2012-05-14

**Authors:** Loes TE Kessels, Robert AC Ruiter

**Affiliations:** 1 Work & Social Psychology, Faculty of Psychology and Neuroscience, Maastricht University, PO Box 616, 6200, MD, Maastricht, The Netherlands

## Abstract

**Background:**

While the majority of the health messages on cigarette packages contain threatening health information, previous studies indicate that risk information can trigger defensive reactions, especially when the information is self-relevant (i.e., smokers). Providing coping information, information that provides help for quitting smoking, might increase attention to health messages instead of triggering defensive reactions.

**Methods:**

Eye-movement registration can detect attention preferences for different health education messages over a longer period of time during message exposure. In a randomized, experimental study with 23 smoking and 41 non-smoking student volunteers, eye-movements were recorded for sixteen self-created cigarette packages containing health texts that presented either high risk or coping information combined with a high threat or a low threat smoking-related photo.

**Results:**

Results of the eye movement data showed that smokers tend to spend more time looking (i.e., more unique fixations and longer dwell time) at the coping information than at the high risk information irrespective of the content of the smoking-related photo. Non-smokers tend to spend more time looking at the high risk information than at the coping information when the information was presented in combination with a high threat smoking photo. When a low threat photo was presented, non-smokers paid more attention to the coping information than to the high risk information. Results for the smoking photos showed more attention allocation for low threat photos that were presented in combination with high risk information than for low threat photos in combination with coping information. No attention differences were found for the high threat photos.

**Conclusions:**

Non-smokers demonstrated an attention preference for high risk information as opposed to coping information, but only when text information was presented in combination with a high threat photo. For smokers, however, our findings suggest more attention allocation for coping information than for health risk information. This preference for coping information is not reflected in current health messages to motivate smokers to quit smoking. Coping information should be more frequently implemented in health message design to increase attention for these messages and thus contribute to effective persuasion.

## Background

An important goal of health education information is to encourage and motivate people to engage in health promoting and disease preventive behaviours. A prerequisite for achieving this goal is that people attend to the persuasive message to which they are exposed to [1,2]. One way to achieve attention for the health information is to explicitly present the severe consequences of risky behaviours performed by the target population, presumably because negative information is believed to attract more attention than positive information [3-5].

Nowadays, cigarette packages frequently include health warnings (e.g., “Smoking causes fatal lung cancer”) with the aim to motivate people to refrain from smoking. A large body of self-report studies suggest that health warning labels are effective in informing people about the negative health consequences of smoking [6-14]. The self-reported cognitive and behavioural impact of health warning labels was found to be largest with prominent labels (i.e., in front and back of the package) supplemented with emotionally graphic warnings that demonstrate the negative bodily impacts or human suffering due to smoking [11-15]. Research on appraisal, recall and level of engagement showed the largest impact when anti-tobacco television ads were presented with ‘visceral negative’ themes [16]. Particularly for starters and smokers intending to quit smoking, graphic warnings that are highly visible and show the negative health consequences of smoking were identified as successful vehicles for reducing smoking prevalence [8]. However, none of these studies used an experimental setup with manipulation of both textual and imagery content. Furthermore, most studies into the effectiveness of graphic health warnings and health communication rely on self-report measures. This runs the risk of false introspection from the participants as people may not be aware of (implicit) motives that drive their behaviour [17-22].

Threatening health information is often used in health messages to increase risk perceptions [23]. In contrast to the findings above, the experimental evidence regarding the effectiveness of threatening health information on measures of protection motivation and behaviour change has been mixed [24-37]. While self-relevant information usually attracts attention from the reader [38,39], a large body of experimental research suggests that people who are most at risk, have the least attention for the risk information, report the least motivation to change, and subsequently react defensively to the message by means of avoidance and denial [21,22,25-27,29-31,40]. Early empirical support for the counterproductive effects of health messages was reported by Liberman and Chaiken (1992)[33]. In an experimental study, they demonstrated that coffee drinkers were less critical of information questioning the link between caffeine and fibrocystic disease and more critical of information supporting the link than non-coffee drinkers [33].

Leading theoretical frameworks on the use of threatening health messages, such as protection motivation theory [41] and the extended parallel process model [42] dictate that for threatening health messages to be effective individuals must feel capable to perform the recommended action (i.e., high self-efficacy) [43]. For a range of health-related behaviours, self-efficacy beliefs have been identified as strong determinants of behaviour change and maintenance [44-48]. In addition, meta-analyses of fear appeal studies have identified self-efficacy and not risk perception as a major predictor of the intention to undertake action to protect health [45,47-51]. While self-efficacy is one of the most widely applied constructs across theories of health behaviour and presenting information that provides help to undertake action to protect health (i.e., coping information) might increase levels of self-efficacy [52], coping information is only rarely presented in health messages in the public domain [53].

The effects of message features and personal characteristics on processes of attention allocation have not been addressed systematically in persuasion research. An exception is formed by three ERP-studies recently conducted by our group that investigated attention allocation processes for health messages that varied in threatening content and self-relevance [21,22,39]. These studies found that individually tailored, non-threatening health communications receive more attention than non-tailored, non-threatening health communications [21,39], whereas threatening as opposed to non-threatening health messages are met with defensive avoidance among those for whom the health threat is personally relevant (i.e., smokers watching high threat smoking pictures) [22].

In the present study, we chose to use the method of eye-movement registration to measure attention allocation processes for health information. Eye-movement registration enables us to measure the course of attention over longer periods of time [37]. Eye movements, as important indicators of visual attention [32,54], compromise a sequence of fixations (i.e., discrete periods of immobility of the eye) and saccades (i.e., quick jumps between fixation locations). During fixating, attention is paid to the stimulus and information is extracted, whereas during saccades vision is basically suppressed [54,55]. In eye-tracking studies the number of fixations is related to the amount of information extracted from a stimulus [54,55]. Besides the number of fixations, dwell time, the amount of time that attention spends at a location once it is deployed to that location [56], can also be used as an indicator of the amount of attention paid to a stimulus [57]. Dwell-time represents the total duration (in milliseconds) of time that was spent looking at a stimulus [37].

The primary purpose of the present study was to examine the amount of attention allocation to risk information and coping texts on cigarette packages. We sought evidence for the hypothesis that people for whom the health risk information is self-relevant will react defensively to this information by spending less attention – reflected in fewer fixations and less dwell time – to high risk as opposed to coping information. We expect that this differential attention response for high risk and coping information will be stronger when the health text is combined with a high threat photo than with a low threat photo because of the increased overall threatening content. For non-smokers, we expect that high risk information might attract more automatic attention than coping information because of the evolutionary value to detect risks (e.g. predator) over benefits (e.g., food) [58,59]. Finally, because coping information contains self-relevant information without any threat information, and self-relevant information attracts attention from the reader [39], we expect that coping texts will attract more attention from smokers than from non-smokers.

## Methods

### Participants and design

In total, 66 Dutch psychology undergraduate students took part in the experiment, 25 daily smoking students (16 women) and 41 non-smoking students (31 women). Normal or corrected-to-normal vision was used as an eligibility criterion. Participants were 18 to 24 years of age (Smokers: *M* = 20.69; *SD* = 1.99, Non-Smokers: *M* = 19.73; *SD* = 1.82). Participants were recruited in various public places at the university campus. They took part for course credits or received a gift voucher of 12 Euros. The experiment varied the content of the health text (high risk vs. coping) and photo (high threat vs. low threat) as within-subjects factors and smoking status (smoker vs. non-smoker) as between-subjects factor. The study was approved by the Ethical Committee Psychology of the School of Psychology and Neuroscience, Maastricht University, that functions in accordance with the Helsinki Declaration.

### Stimulus materials

The stimulus materials were sixteen self-created pictures of cigarette packages (size, H × W: 11.22 × 7.87 in.). We used the basic format of a cigarette package and added a smoking related photo and health text. The upper part of each cigarette package was held constant, with the texts ‘cigarettes’ and ‘brand’ printed in a red and a white rectangle, respectively. The upper part of the package (‘cigarettes’ and ‘brand’) was 50% of the total picture (H × W: 5.61 × 7.87 in.). In the middle part of each cigarette package one of sixteen different low threat (8) and high threat (8) smoking-related coloured photos was presented (40% of the total picture; H × W: 4.53 × 7.87 in.). Below the picture we varied the content of the health texts (10% of the total picture; H × W: 1.10 × 7.87 in.). In total, we presented sixteen smoking-related health texts in white letters on a black background.

The health texts and photos on the cigarette packages were selected from two pilot studies. In the first pilot study, we tested twenty-four smoking-related health texts that were partly based on existing texts on cigarette packages (http://www.tobaccolabels.ca/). Sixteen smoking and fifteen non-smoking undergraduate students (not tested for main study) rated each text on 7-point scales measuring whether the text contains helpful information regarding quitting smoking (1 = *not at all*, 7 = *very much*) and the *perceived level of threat* of the text (‘This photo is threatening’; 1 = *not at all,* 7 = *very much)*. Eight coping texts and eight high risk texts were selected. The selected coping texts were believed to contain more helpful information about quitting smoking (*M =* 3.50; *SD* = 1.21) than the high risk texts (*M =* 1.64; *SD =* .84), *t*(30) = 6.69, *p* < .001, whereas the high risk texts were evaluated as more threatening (*M =* 2.09; *SD* = .63) than the coping texts (*M* = 1.58; *SD* = .50), *t*(30) = 4.99, *p* < .001. An example of the selected coping texts is “You can do it, your doctor or pharmacist can help you stop smoking”. An example of the high risk texts is “Smoking clogs the arteries and causes heart attacks and strokes”.

In the second pilot study, thirty-five smoking-related photos were tested that were found on the website of the European Community presenting the cigarette packages and via a search through the internet using search terms like ‘smoking’, ‘cigarette’, or ‘causes smoking’. On a 7-point scale sixteen smoking and sixteen non-smoking undergraduate students (not tested for main study) rated the *perceived level of threat* (‘This photo is threatening’; 1 = *not at all,* 7 = *very much)* for each photo. We made a selection of eight low threat photos with a maximum score of 2 on the perceived level of threat scale and eight high threat photos with a minimum score of 3.5 on the perceived level of threat scale. The high threat photos contained pictures with black lungs, skeletons with a smoking cigarette and other images that illustrate the negative health consequences of smoking. The low threat pictures showed smoking cigarettes, people smoking a cigarette, and other images that do not directly focus on the negative health consequences of smoking.

For creating the sixteen cigarette packages for the main experiment each low threat and high threat photo was paired to one risk or coping text with the result that each of the sixteen images used to represent a cigarette package contained a unique combination of photo and health text (risk or coping text). Within each text*photo condition four combinations were presented. All other features of the cigarette packages (e.g., colour, font type) were kept constant across conditions. The final set of stimulus materials is available from the first author on request.

### Apparatus

Eye movements were registered by the EyeLink I eyetracker from SensoMotoric Instruments (SMI, Germany) and SR Research (Canada), with a 250 Hz temporal resolution, a 0.005˚ gaze and eye position resolution, and a gaze position accuracy with 0.5−1.0˚ average error. It is a headband-mounted infrared video-based tracking system that can track both eyes. The system corrects for head-motion by means of an additional infrared camera.

The participant’s head rested on a chin-rest at an approximate distance of 57 cm of the computer monitor (19” flat panel Dell monitor). Monitor resolution was set to 1024 * 768 pixels. The cigarette packages were presented in colour format and sized 7 by 13 cm. The presentation of stimuli and analysis of the eye tracking was performed with Matlab 6.5. Although viewing was binocular, only fixations and saccades of the right eye were monitored.

### Procedure

The experiment took place in a dimly lit, sound-attenuating room. After explaining the procedure of the experiment, participants signed an informed consent, and the Eyelink system was installed on the head. Participants were seated at a table in front of a computer screen, with their head resting on a chin-rest. After calibrating and validating the Eyelink system, the experiment started.

Each of the sixteen cigarette packages was randomly presented for 10 seconds. Before a package appeared on the screen, a dot was presented at the centre of the screen. Participants were asked to focus on the dot, so that each participant would start viewing every package from the same position on the screen. During exposure to the stimuli, eye movements were registered. After presentation of the sixteen different packages, the headset was removed and the participant started with filling out the self-report questionnaires to evaluate the stimuli.

### Eye movement registration

We extracted the eye tracking data from Data Viewer (SR Research software) to SPSS. For analysing the eye-movements we created two areas of interests (i.e. two rectangles) for each cigarette package. The first area of interest contained the health text (coping vs. high risk) and the second area of interest contained the photo. The number of fixations and dwell time for the area of interest of each health text were divided by the number of syllables per text. The mean number of syllables was not different across conditions, *F*s < 1.53, *ns*, η_p_^2^ < .05, (coping text – low threat photo: *M* = 14.5; *SD* = 3.11; range: 12–19; high risk text – low threat photo: *M* = 14.5; *SD* = 1.91; range: 12–16; coping text – high threat photo: *M* = 14.75; *SD* = 3.59; range: 12–20; high risk text – high threat photo: *M* = 16; *SD* = 5.35; range: 10–21).

Finally, for each area of interest we compared the number of fixations and dwell time for all different conditions after averaging for each condition the responses to the four stimuli within that condition.

### Self-report measures

To measure participants’ evaluation of the health texts and the photos, we used self-report items with 7-point Likert scales. Five items that measured clarity, credibility, interest, usefulness and acceptability of each of the presented risk and coping texts were combined to measure message acceptance of the health texts (α’s > .88; 1 *= not at all*, 7 = *very much*). Novelty and perceived personal relevance of the text were both measured with one item (1 *= not at all*, 7 = *very much*). Furthermore, *perceived level of threat* was assessed for each health text and each photo (‘This text/photo is threatening’; 1 = *not at all,* 7 = *very much)*.

### Data-analysis

Mixed analysis of variances (ANOVA) were conducted to test the effects of within-subjects factors *health text* (coping vs. high risk) and *photo* (low threat vs. high threat) and the between-subjects factor *smoking status* (smokers vs. non-smokers) on the number of fixations and dwell time. Similar analyses were conducted for the self-report measures. Significant interactions between health text, photo and smoking status were followed-up by mixed analyses of variance (ANOVA) for each level of photo. To control for sphericity violations in the mixed ANOVAs, we report probability values with Greenhouse-Geisser correction for *F* tests with more than one degree of freedom in the numerator. The reported estimates of the effect size are the partial eta squared (η_p_^2^) for the analyses of variance. Only significant effects are reported (*p* < .05), whereas lower order effects in the omnibus analyses are only reported in case no significant higher order effects were found. In case of main effects of health text, photo and smoking status overall means are provided in the text.

## Results

### Sample characteristics

No differences between smokers and non-smokers were observed in terms of age and gender, *p*s > .06 (see Table 1 for demographics). Smoking participants smoked somewhat more than six cigarettes per day on average (*M* = 6.40*; SD* = 4.30). The data sets of two smoking participants could not be used because of technical problems. Therefore 64 participants remained in the analyses.

**Table 1:** Eye movements (fixations and dwell time) for AOIs by smoking status – text and photo

	**High Threat Photo**				**Low Threat Photo**			
	Coping		High Risk		Coping		High Risk	
	Smokers	Non-Smokers	Smokers	Non-Smokers	Smokers	Non-Smokers	Smokers	Non-Smokers
AOI Text								
Fixations	0.82	0.74	0.71	0.82	0.86	0.86	0.70	0.69
	(0.22)	(0.27)	(0.23)	(0.27)	(0.31)	(0.29)	(0.25)	(0.21)
Dwell-Time	179.04	154.93	147.89	171.09	187.57	181.08	150.40	141.08
	(42.87)	(54.62)	(60.20)	(59.46)	(75.74)	(66.13)	(62.43)	(44.54)
AOI Photo								
Fixations	16.84	17.57	17.41	16.83	13	14.72	16.95	17.29
	(4.91)	(4.35)	(4.94)	(4.06)	(4.15)	(3.95)	(5.02)	(3.78)
Dwell-Time	5074.52	5365	5242.72	4989.24	4442.04	4593.24	5380.61	5496.34
	(1151.73)	(1304.78)	(979.91)	(1153.33)	(928.38)	(1098.40)	(873.79)	(1149.38)

Note. Smokers (n = 23); Non-Smokers (n = 41)

Means (SD) of fixations and dwell Time (in milliseconds) for the AOIs text and photo for each health text (coping vs. high risk) in combination with a high threat photo or a low threat photo.

### Eye tracking

Table 1 shows the mean number of fixations and dwell-time for each area of interest (health text and photo), for each experimental condition, and for the two smoking status groups.

#### *Area of interest – health text*

*Fixations.* The number of fixations per syllable for the health texts revealed a significant 3-way interaction between health text, photo and smoking status, *F*(1, 62) = 8.38, *p* < .01, η_p_^2^ = .12.

Separate analyses for each level of photo showed for the health messages combined with a high threat photo a significant interaction effect between health text and smoking status, *F*(1, 62) = 11.00, *p* < .01, η_p_^2^ = .15. Paired-samples t-tests for each level of smoking status showed the expected patterns of results. Smokers showed more fixations for coping information than for high risk information, *t*(22) = 2.37, *p* < .05. For non-smokers, more fixations were found for high risk information than for coping information, *t*(40) = −2.41, *p* < .05.

In the low threat photo conditions a significant main effect of health text was found, *F*(1, 62) = 51.04, *p* < .001, η_p_^2^ = .45. For smokers and non-smokers, coping information (*M* = 0.86; *SD =* 0.30) received more fixations than high risk information (*M* = 0.70; *SD =* 0.22).

*Dwell time.* A significant 3-way interaction between health text, photo, and smoking status was also found on the average dwell time per syllable of the health texts, *F*(1, 62) = 7.63, *p* < .01, η_p_^2^ = .11. Separate analyses for each level of photo showed for the high threat photo conditions a significant interaction effect between health text and smoking status, *F*(1, 62) = 11.92, *p* < .01, η_p_^2^ = .16. Paired-samples t-tests for each level of smoking status showed the expected patterns of results with more dwell time for coping information than for high risk information for smokers, *t*(22) = 2.68, *p* < .05, and more dwell time for high risk information than for coping information for non-smokers, *t*(40) = −2.04, *p* < .05.

For the low threat photo conditions again a significant main effect of health text was found, *F*(1, 62) = 44.27, *p* < .001, η_p_^2^ = .42. Coping information (*M* = 183.41; *SD =* 69.21) received more dwell time than high risk information (*M* = 144.43; *SD =* 51.39) in both smokers and non-smokers.

#### *Area of interest – photo*

*Fixations.* The average number of fixations for the area of interest of the photo showed significant 2-way interactions between health text and photo, *F*(1, 62) = 22.46, *p* < .001, η_p_^2^ = .27, and between health text and smoking status, *F*(1, 62) = 5.65, *p* < .05, η_p_^2^ = .08. Separate analyses for each level of photo showed for the high threat photos no significant effects involving the factors smoking status or health text, *F*s < 1.89, *ns*, η_p_^2^ < .03. For the low threat photos a significant effect of health text was found, *F*(1, 62) = 58.90, *p* < .001, η_p_^2^ = .49. Low threat photos presented with high risk information (*M =* 17.17; *SD* = 4.23) received significantly more fixations than low threat photos presented with coping information (*M* = 14.10; *SD* = 4.07).

*Dwell time.* On the dwell time for the photos only a significant 2-way interaction between health text and photo was found, *F*(1, 62) = 35.09, *p* < .001, η_p_^2^ = .36. Separate analyses for each level of photo showed for the high threat photos no significant effects involving the factors smoking status or health text, *F*s < 3.88, *ns*, η_p_^2^ < .06. For the low threat photos a significant main effect of health text was found, *F*(1, 62) = 74.07, *p* < .001, η_p_^2^ = .54. Low threat photos presented with high risk information (*M* = 5454.70; *SD =* 1052.87) received significantly more dwell time than low threat photos presented with coping information (*M* = 4538.91; *SD =* 1035.54).

### Self-report measures

An overview of the mean scores on the measures of message acceptance, novelty, personal relevance and perceived threat of the health texts by smokers and non-smokers can be found in Table 2.

**Table 2:** Self-report Measures for texts and photos by smoking status

	**High Threat Photo**				**Low Threat Photo**			
	Coping		High Risk		Coping		High Risk	
	Smokers	Non-Smokers	Smokers	Non-Smokers	Smokers	Non-Smokers	Smokers	Non-Smokers
Text								
Acceptance	4.25	4.95	5.06	4.97	4.32	4.94	5.24	5.24
	(1.32)	(1.00)	(0.83)	(1.14)	(1.17)	(1.04)	(0.94)	(1.11)
Novelty	3.16	3.57	3.20	3.41	3.28	3.68	3.04	3.35
	(1.69)	(1.28)	(1.50)	(1.41)	(1.52)	(1.21)	(1.12)	(1.38)
Relevance	2.35	1.23	3.11	1.21	2.45	1.20	2.76	1.37
	(1.14)	(0.70)	(1.33)	(0.66)	(1.19)	(0.65)	(1.20)	(0.98)
Threat	1.73	1.69	4.09	3.87	1.82	1.53	4.02	3.99
	(0.97)	(0.84)	(1.25)	(1.59)	(1.02)	(0.86)	(1.23)	(1.68)
Photo								
Threat	4.49	4.38	4.74	4.58	1.72	1.51	1.87	1.58
	(1.29)	(1.47)	(1.27)	(1.76)	(1.02)	(0.78)	(1.12)	(1.05)

Note. Smokers (n = 23); Non-Smokers (n = 41)

Means (SD) of self-report measures for message acceptance, novelty, relevance and threat for each health text (coping vs. high risk) in combination with a high threat photo or a low threat photo. Message evaluation, novelty and relevance was only measured for the AOIs **text,** perceived threat level was measured for the AOI **Text** and the AOI **Photo.**

*Message acceptance.* A significant interaction between health text and smoking status was found on message acceptance, *F*(1, 62) = 10.52, *p* < .01, η_p_^2^ = .15. Separate analyses for each level of smoking status showed that smokers evaluated high risk information (*M* = 5.15; *SD =* 0.85) more positively than coping information (*M* = 4.29; *SD =* 1.19, *t*(22) = −4.62, *p* < .001). For non-smokers, no significant difference was found between high risk information (*M* = 5.10; *SD =* 1.10) and coping information (*M* = 4.94; *SD =* 1.00, *p* = .21).

*Novelty.* No significant main effects or interaction effects were found on the extent to which participants evaluated the presented texts as novel, *F*s < 1.69, *ns*, η_p_^2^ < .03.

*Personal Relevance.* A significant 3-way interaction between health text, photo, and smoking status was found on the perceived personal relevance of the health texts, *F*(1, 62) = 7.93, *p* < .01, η_p_^2^ = .11. Separate analyses for each level of photo showed for health texts combined with a high threat photo a significant interaction between health text and smoking status, *F*(1, 62) = 16.20, *p* < .001, η_p_^2^ = .19. Smokers judged the high risk information (*M* = 3.11, *SD* = 1.33) as more personally relevant than the coping information (*M* = 2.35, *SD* = 1.14, *t* (22) = −3.09, *p* < .01). For non-smokers no difference was found between high risk information (*M* = 1.23, *SD* = .70) and coping information (*M* = 1.21, *SD* = .66, *t* (40) = .43, *p* = .67).

For health texts combined with a low threat photo, significant main effects of health text, *F*(1, 62) = 7.18, *p* < .01, η_p_^2^ = .10, and smoking status, *F*(1, 62) = 30.86, *p* < .001, η_p_^2^ = .33, were found. High risk information (*M* = 1.87, *SD* = 1.25) was judged to be more personally relevant than coping information (*M* = 1.64, *SD* = 1.06). In addition, smokers (*M* = 2.60, *SD* = 1.10) judged the health information as more personally relevant than non-smokers (*M* = 1.28, *SD* = .0.79).

*Threat.* A significant main effect of health text was found on perceived threat, *F*(1, 62) = 157.93, *p* < .001, η_p_^2^ = .72. High risk information (*M* = 3.97; *SD =* 1.40) was judged to be more threatening than coping information (*M* = 1.67; *SD =* 0.87). The main effect of photo was also significant, *F*(1, 62) = 370.06, *p* < .001, η_p_^2^ = .86. High threat photos (*M* = 4.53; *SD =* 1.40) were judged to be more threatening than low threat photos (*M* = 1.63; *SD =* 0.85).

## Discussion

The present study examined whether smokers (but not non-smokers) react defensively to smoking-related risk information by spending less attention to high risk information than to coping information. Indeed, in smokers we found more fixations and longer dwell time in response to coping information as opposed to high risk information, irrespective of the threat level (high vs. low) of the accompanying smoking-related photos. This finding supports the importance of adding instructions about how to effectively adopt healthy behaviour to increase attention for health communications [60,61].

For non-smokers, an attention preference was found for high risk information above coping information, but only when the health information was combined with a high-threat photo. As young non-smokers are often a key target group for pictorial warning labels, the increased attention for high risk information in combination with high threat photos suggests the importance of adding pictorial warnings for impeding smoking uptake and promoting non-smoking norms in non-smoking populations [13,18]. The findings for smokers and non-smokers together further support the hypothesis that threatening health information attracts the least attention among those to whom the health threat is most personally relevant [22,40]. At the same time, studies are needed that examine the extent to which decreased attention for self-relevant high risk information transfers to positive effects of health messages with regard to the prevention of smoking uptake and the promotion of smoking cessation.

Contrary to our expectations based on the evolutionary value of detecting risk in an early stage of information processing, and the finding that non-smokers judged the materials as less personally relevant than smokers, smokers and non-smokers did not differ on the amount of fixations and length of dwell time for the two types of texts when these were presented in combination with low threat photos. Instead both smokers and non-smokers, allocated more attention to coping information than to high risk information. In addition, with respect to the eye-movement data for the photos, smokers and non-smokers did not allocate attention differently to high threat and low threat photos. The results did show however that both groups had more fixations and longer dwell time for low threat photos presented with a high risk text than for low threat photos presented with a coping text. No attention differences were found for the high-threat photos in combination with either high risk or coping information. Overall, these secondary findings suggest that a high-threat photo seems to have a stronger impact on directing attention allocation processes for written health messages on a cigarette package than a low-threat photo. Similar to one of our previous studies [22], high-threat images seem to attract people’s attention, but subsequent allocation processes are dependent on the self-relevance of the textual information with more attention for risk information among non-smokers and more attention for coping information (or more disengagement with risk information) among smokers.

The finding that coping text information received more attention than high risk text information in smokers does not seem to be the result of the novelty of the presented textual information. Although coping information is much less visible on cigarette packages in the EU than threat information – 12 out of 14 health texts containing high risk information and only two texts containing coping information – smokers (and also non-smokers) did not judge the coping information to be more novel than the high risk information. In many countries outside Europe, risk and coping information are frequently combined in a single message. Future research should investigate whether this type of mixed messaging works better than either approach by itself.

A potential weakening of the conclusion regarding the greater importance of coping information above threat information among smokers is the finding that smokers judged the coping information as less positive and less personally relevant than the high risk information. This finding is in line with previous research showing that photos of people smoking (like those we used as low threat photos) are rated more positively by smokers than photos without smoking scenes [12]. A possible explanation could be that because the coping information received most attention, the viewers had more time to be critical on the information and subsequently judged the information as less positive and less personally relevant than the risk information. Another explanation is that risk information is simply more imminent than coping information because referring directly to personally relevant threats and therefore is more positively evaluated and considered to be more personally relevant. Despite these differences it is important to note that coping information was evaluated positively rather than negatively with mean scores situated about the above scale midpoints. Finally, participants may have responded with social desirable statements. This study thus provides further evidence for the need to use more direct measures when studying important psychological processes that are conditional to the effectiveness of health communications [20-22].

The present study has some limitations. First, we did not include a control text with neutral smoking information (e.g., “cigarette packages contain at least 19 cigarettes”). Although a comparison between neutral versus coping or high risk information would be useful to determine whether coping information results into more attention or less defensive avoidance compared with high risk information in smokers, neutral smoking texts would be at odds with the goal of the health messages and could therefore raise questions about the relevance and quality of this information and thus may interfere with attention processes under study. Second, we did not measure any recall or behaviour effects of the presented health texts. Consequently, we cannot make any assumptions about an association between attention and memory or between attention and behaviour for the presented information. It should be noted however that within-subjects designs are preferred in attention research to control for often strong inter-individual differences. At the same time, these designs make comparisons between experimental conditions at the level of recall and behaviour complicated due to possible contamination of experimental conditions. Another limitation is that attention was measured under conditions of forced exposure to novel stimuli. Although previous ERP attention studies already showed defensive reactions towards repeatedly presented high threat health information that is personally relevant [21,22], future research should investigate eye movement responses for initial and repeated exposure to warning label messages. Finally, although we tested smokers that did not made any attempts to quit smoking, there are different kinds of smokers that could respond differently to the different messages. For example, the coping messages may have their greatest impact among smokers who are intending to quit.

## Conclusions

By recording eye movements, the present study finds evidence that written coping information presented on cigarette packages attracts more attention from the viewer than high risk textual information, especially for those for whom the information is self-relevant (i.e., smokers). For non-smokers, for whom the health information is not self-relevant findings show mixed results, with attention preferences for coping information only when the information is combined with a low threat photo. This finding is in line with meta-analysis studies into the effectives of behavioural change interventions in other health domains such as HIV/AIDS and nutrition and physical activity [60,61]. These studies show that providing instructions about how to effectively adopt healthy behaviour could be a more useful tool in health education message design than providing threat information about the negative consequences of unhealthy behaviour X. The findings of the present study support this conclusion.

## Competing interests

The authors declare that they have no competing interests.

## Authors' contributions

LTEK carried out the eye-movement study, the analyses and drafted the manuscript. RACR participated in the design of the study, supervised the study conduct and helped to draft the manuscript. All authors read and approved the final manuscript.
